# IL-2 immunotherapy for targeting regulatory T cells in autoimmunity

**DOI:** 10.1038/s41435-023-00221-y

**Published:** 2023-09-23

**Authors:** Valentina Lykhopiy, Vanshika Malviya, Stephanie Humblet-Baron, Susan M. Schlenner

**Affiliations:** 1 Department of Microbiology, Immunology and Transplantation, KU Leuven-University of Leuven, Leuven, Belgium; 2 argenx BV, Industriepark Zwijnaarde 7, 9052 Ghent, Belgium

**Keywords:** Immunosuppression, Translational immunology

## Abstract

FOXP3^+^ regulatory T cells (T_reg_) are indispensable for immune homoeostasis and for the prevention of autoimmune diseases. Interleukin-2 (IL-2) signalling is critical in all aspects of T_reg_ biology. Consequences of defective IL-2 signalling are insufficient numbers or dysfunction of T_reg_ and hence autoimmune disorders in human and mouse. The restoration and maintenance of immune homoeostasis remain central therapeutic aims in the field of autoimmunity. Historically, broadly immunosuppressive drugs with serious side-effects have been used for the treatment of autoimmune diseases or prevention of organ-transplant rejection. More recently, ex vivo expanded or in vivo stimulated T_reg_ have been shown to induce effective tolerance in clinical trials supporting the clinical benefit of targeting natural immunosuppressive mechanisms. Given the central role of exogenous IL-2 in T_reg_ homoeostasis, a new and promising focus in drug development are IL-2-based approaches for in vivo targeted expansion of T_reg_ or for enhancement of their suppressive activity. In this review, we summarise the role of IL-2 in T_reg_ biology and consequences of dysfunctional IL-2 signalling pathways. We then examine evidence of efficacy of IL-2-based biological drugs targeting T_reg_ with specific focus on therapeutic candidates in clinical trials and discuss their limitations.

## Introduction

In 1976, the supernatant of activated T cells was found to contain a potent T cell growth factor, which was cloned in 1983 as interleukin-2 (IL-2) [1–3]. The identification of IL-2 marked the start of substantial efforts to unravel IL-2-dependent immunological processes, to mechanistically understand IL-2 binding to its receptor and to dissect the signalling pathways downstream of receptor activation. Importantly, with the discovery of IL-2 and an increasing knowledge on IL-2 functions, immense research efforts were launched to develop IL-2-based immunotherapies to exploit its properties in cancer and autoimmune diseases. Here, we provide a brief overview on IL-2 signalling, its relevance in the biology of regulatory T cells (T_reg_), and detail recent advances in IL-2-based immunotherapeutics for autoimmune and inflammatory diseases predominantly in clinical stages of development.

## IL-2 expression, capture and signalling

The signalling-competent IL-2 receptor (IL-2R) is expressed either as heterodimer or -trimer [4]. The dimeric IL-2R consists of the IL-2Rβ chain (CD122, shared with IL-15R) and the common γ chain (γ_c_, CD132, shared with the receptors for IL-4/7/9/15/21) [4–10] and displays intermediate affinity for IL-2 (Kd~10^−9^ M). It can hence signal upon binding of IL-2 as well as IL-15. The trimeric IL-2R additionally includes the IL-2Rα chain (CD25) [11]. CD25 can be considered a monomeric IL-2R as it binds IL-2, however, it is not capable of signalling. Although CD25 itself displays only low affinity (Kd~10^−8^ M) and a high on-off rate for IL-2, it delivers IL-2 to the dimeric receptor [11] and its presence increases the affinity of the trimeric receptor for IL-2 100-fold (Kd~10^−11^ M), consequently providing the expressing cells with a substantial competitive advantage in IL-2 capture [12–14].

Various immune and non-immune cell types express the IL-2R. In humans and mice, the dimeric IL-2R is expressed at low levels by CD4 memory T cells and naïve T cells and at high levels by CD8 memory T cells [15, 16] and CD56^low^ NK cells [17]. In mice, the trimeric IL-2R on the other hand is expressed highest on T_reg_ [18, 19] and at lower levels on recently activated and effector CD8 T cells, ILC2, and some NKT and CD56^bright^ NK cells [17, 20–23]. Similarly, in human peripheral blood mononuclear cells (PBMC), T_reg_ express the highest levels of the trimeric IL-2R whereas other immune cells such as CD45RO^pos^ CD4 T cells, most CD56^high^ NK cells, few CD4 and CD8 naïve T cells express it at lower levels [24]. The trimeric IL-2R is also expressed by endothelial cells, with further CD25 upregulation upon IL-2 treatment [25, 26], and signalling can cause the vascular leak syndrome—a known adverse effect of high-dose IL-2 therapy in mice and patients.

**Fig. 1:**
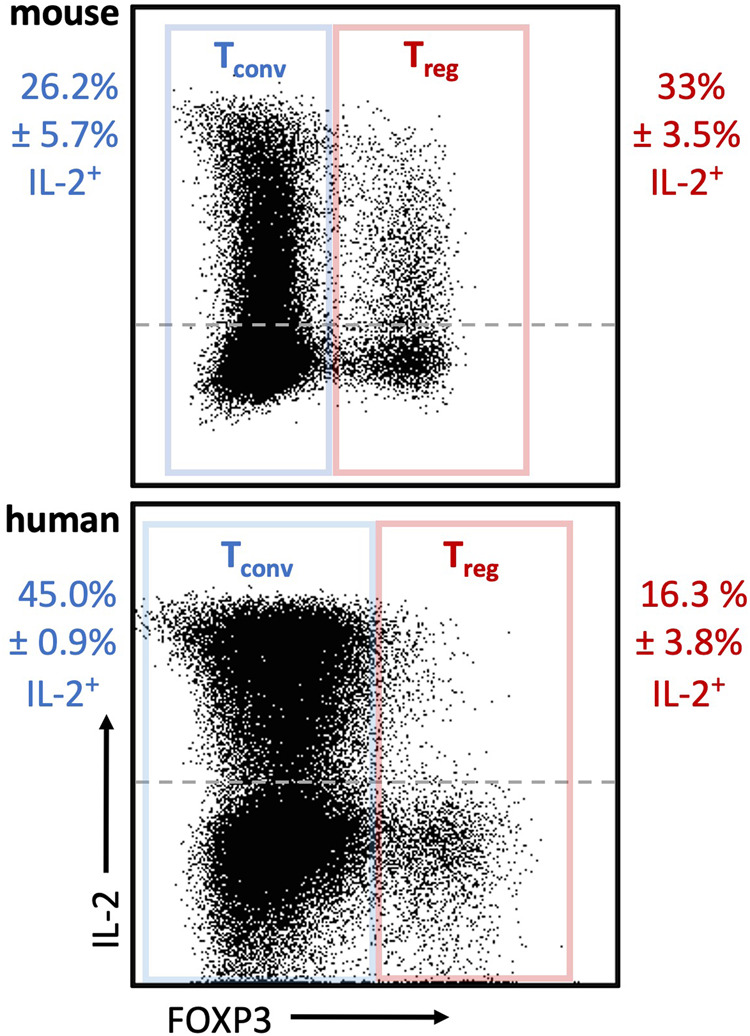
IL-2 expression in mouse and human conventional and FOXP3^+^ CD4 T cells. Mouse splenocytes (wildtype C57Bl6/J, *n* = 4) and healthy human PBMC (*n* = 5) were stained for CD3, TCRγδ [mouse], CD4, FOXP3 and IL-2 along with a viability dye. Representative flow plots depicting IL-2 expression in mouse (top) and human (bottom) conventional T cells (T_conv_, blue) (live CD3^+^ TCRγδ^neg^ [mouse] CD4^+^ FOXP3^neg^) and T_reg_ (red) (live CD3^+^ TCRγδ^neg^ [mouse] CD4^+^ FOXP3^+^). The frequency of IL-2^+^ cells of FOXP3^neg^ and FOXP3^+^ cells is shown (mean ± SEM). The geometric mean fluorescence intensities of IL-2 as a measure to compare per cell protein levels between T_conv_ and T_reg_ are 2958 ± 160 (mouse T_conv_) vs 2130 ± 125 (mouse T_reg_) and 7308 ± 904 (human T_conv_) vs 5555 ± 452 (human FOXP3^pos^ cells) (mean ± SEM). Ethical approvals were obtained from the KU Leuven Animal Ethics Committee (150/2019) and the University Clinic Leuven Ethical Committee (S65883). Antibodies were purchased from BD Biosciences (564667, 566405, 624295), Biolegend (100225, 503840, 320214), Miltenyi Biotec (130–111–601), and ebioscience (65-0865-18, 56-0038-80, 48-0048-42).

IL-2 is a pleiotropic cytokine that can act in an autocrine and paracrine way, with cell type- and context-dependent positive effects on survival, population expansion or lineage stability [17, 27–29]. The main source of IL-2 are CD4 conventional T cells upon T cell receptor (TCR)/CD28 (co-) stimulation [30, 31]. Other immune cells such as CD8 T cells, NK(T) cells or dendritic cells can produce IL-2 as well albeit at lower quantities [24, 29]. T_reg_ are highly dependent on exogenous IL-2 sources as FOXP3 in cooperation with other transcription factors represses *Il2* transcription [32–34]. Yet, a sizable population of T_reg_ in mice is capable of producing IL-2, albeit at a lower per cell level compared to Foxp3^neg^ CD4 T cells [29]. In contrast, IL-2 expression in human peripheral blood FOXP3^pos^ CD4 T cells is limited to a subset of cells with low expression of FOXP3 likely not representing suppressive T_reg_ (Fig. 1) [35].

**Fig. 2:**
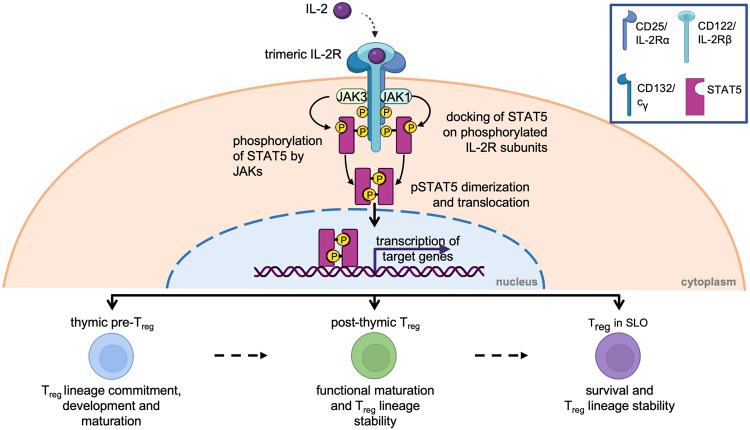
Critical roles of IL-2 in T_reg_. Upon binding of IL-2 to the trimeric IL-2R, JAK1 and JAK3 phosphorylate the IL-2Rβ or IL-2R common γ (c_γ_) chain, respectively. STAT5 docks onto the phosphorylated residues and is then phosphorylated by JAK1/3. Phosphorylated STAT5 (pSTAT5) dimerises and translocates to the nucleus to bind its target loci (such as *FoxP3*/*FOXP3*). IL-2 signalling is critical in T_reg_ biology. It plays a dominant role in thymic T_reg_ development (bottom, left), during peripheral T_reg_ functional maturation in barrier tissues (bottom, middle), and is indispensable for the survival and functional lineage stability of mature T_reg_ in secondary lymphoid organs (SLO) (bottom, right).

Upon binding of IL-2 to its receptor the quaternary complex is internalised, CD25 is recycled back to the cell surface while CD122 and CD132 are degraded [36, 37]. The IL-2:IL-2R complex can signal via three major pathways, each activating different downstream transcriptional regulators [27, 38, 39]. Depending on the downstream signalling pathway, PI3K (PI3K-AKT-mTOR pathway), SHC1 (MAPK pathway) or STAT (signal transducer and activator or transcription) 5 (Janus activating kinase [JAK]1/3-STAT pathway) are triggered. In T cells, and particularly T_reg_, phosphorylation of the IL-2Rβ chain and common γ chain by JAK1 and JAK3 and subsequent activation of STAT5 accounts for 90% of IL-2 signalling (Fig. 2)[40, 41]. In T_reg_, the PI3K-AKT-mTOR pathway is suppressed by PTEN (phosphatase and tensin homologue). This mechanism regulates T_reg_ homoeostasis by negatively regulating proliferation and positively regulating lineage stability likely by increased nuclear translocation of FOXO1/FOXO3a [42–44]. Further, to regulate T_reg_ homoeostasis and maintain T_reg_ lineage fate, STAT5 activation and SOCS1 expression regulate each other in a positive inhibitory loop. IL-2 signalling induces SOCS1 expression and SOCS1 in turn attenuates IL-2R signalling by blocking JAK proteins, hence interrupting the phosphorylation of STAT5 [45–49].

T_reg_ homoeostasis is essential to preserve the delicate balance of immune activation. Absence of T_reg_ or decreased function will result in autoimmune diseases, while abundance of T_reg_ will lead to overt immune suppression. These events are balanced by the exclusive IL-2 capture sensitivity of T_reg_, an overall high dependency of T_reg_ on exogenous IL-2 sources and, hence, a reciprocal control between effector T cells and T_reg_. The preferential and high expression of the trimeric IL-2R renders T_reg_ most sensitive to IL-2 capture thereby outcompeting other cell types. This superior efficiency in IL-2 capture is exploited by low-dose IL-2 therapy to specifically target and expand T_reg_ [50, 51]. Further, in T_reg_ the cooperation of the trimeric receptor and the serine/threonine phosphatase PP2A confers increased sensitivity to IL-2 [52, 53] and PP2A deficiency in T_reg_ results in autoimmunity [54]. Consequently, 10-fold lower IL-2 levels are required for STAT5 activation in T_reg_ compared to CD25-expressing non-T_reg_ and optimal IL-2-dependent gene expression in T_reg_ occurs at 100-fold lower IL-2 concentrations compared to other cell types expressing CD25 [55]. The high sensitivity to IL-2 signalling allows for sufficient signalling when available CD25 surface levels are reduced [56]. If the superior IL-2 capture is strongly compromised such as it is in CD25-deficient mice or in patients with risk alleles for CD25, systemic inflammation and/or autoimmunity are the consequence of the resulting T_reg_ deficiency or disturbed T_reg_ homoeostasis.

## IL-2 in T_reg_ biology and function

Initially, and with the assumption that the main function of IL-2 was the activation of effector T cells and NK cells, efforts to exploit IL-2 in immunotherapy were focused on promoting anti-tumour immunity [57]. High-dose recombinant IL-2 (aldesleukin; trade name Proleukin) was the first immunotherapy approved by the U.S. Food and Drug Administration (FDA) in 1992 [58, 59]. The activation of effector T cells as the main function of IL-2 was contested when ablation of *Il2*, *Il2ra* and *Il2rb* expression in mice caused lethal lymphoproliferation and autoimmunity, rather than immunodeficiency [60]. Ten years later, these observations were explained with the discovery of T_reg_ as an immunosuppressive CD4^pos^ T cell subset characterised by high levels of CD25 and a non-redundant function for IL-2 in many aspects of T_reg_ biology [18, 61]. The absence or dysfunction of T_reg_ results in fatal multiorgan autoimmunity in mice (scurfy [62]) and human (immune dysregulation, polyendocrinopathy, enteropathy, X-linked syndrome, IPEX [63]), and their reduced function has been reported in several systemic (auto-)inflammatory diseases [51, 64–69].

IL-2 signalling (via JAK-STAT5) has been demonstrated to be important for T_reg_ thymic development, peripheral induction, lineage commitment and stability sustainability, and homoeostasis (Fig. 2). T_reg_ development takes place in the thymus (thymic T_reg_, tT_reg_) but conventional CD4 T cells can convert into T_reg_ upon tolerogenic stimulation in the periphery as well (peripherally-induced T_reg_, pT_reg_). IL-2 signalling is important in establishing the T_reg_ identity alongside with TCR and TGFβ signalling [70, 71]. For tT_reg_ development, a two-step model of TCR and cytokine signalling has been proposed in which the main driving cytokine is IL-2 and its induction of the JAK-STAT5 signalling pathway. IL-7 and IL-15 can compensate for the lack of IL-2 but in the presence of IL-2 their receptors are downregulated establishing a dominant role for IL-2 [72–75]. Defective expression of IL-2 or its receptor subunits, caused by single nucleotide polymorphisms in human or via introduced genetic modification in mice, results in a lack of functional T_reg_ and consequently lymphoproliferation, multiorgan infiltration of activated lymphocytes and lethal autoimmunity [15, 60, 76–81]. Similarly, inappropriate regulation of IL-2 signal transduction impairs T_reg_ homoeostasis and functional stability [42, 54, 82–86]. Although genetic studies using germline deletion cannot ultimately dissect the requirement for IL-2 during (thymic or peripheral) T_reg_ development from the requirement for IL-2 during peripheral survival and expansion, several lines of evidence support both intrathymic and peripheral roles for IL-2 in T_reg_.

### IL-2- or IL-2R-deficient mice

Autoimmunity in IL-2- or IL-2R-deficient mice can be prevented by adoptive transfer of T_reg_ demonstrating that proficiency in IL-2-signalling in mature T_reg_ is sufficient and necessary for peripheral tolerance even when thymic T_reg_ development is impaired [61, 87–89]. Notably, T_reg_ numbers but not their suppressive activity can be rescued in IL-2- or CD25-deficient mice by depletion of the pro-apoptotic protein Bim [90]. Also in IL-2-sufficient mice, Bim has been shown to mediate T_reg_ apoptosis to regulate T_reg_ numbers. A critical role for IL-2 in T_reg_ peripheral survival is to maintain the pro-survival protein Mcl-1 [91].

### Antibody-mediated neutralisation or preferential delivery of IL-2

Further, antibody-mediated neutralisation of IL-2 and studies utilising IL-2:anti-IL-2 immune complexes have illustrated the indispensable role of IL-2 in peripheral T_reg_ maintenance and functional maturation. Neutralisation of IL-2 induces T cell-mediated autoimmunity by selectively reducing T_reg_ numbers [92]. Conversely, the application of IL-2:anti-IL-2 immune complexes can prevent the binding of IL-2 to effector T cells and preferentially deliver it to T_reg_ to substantially expand these [93].

### Competitive advantage of IL-2R^WT^ cells in mixed bone marrow chimeras

In T_reg_, IL-2-STAT5 signalling is sensed via the conserved non-coding sequence 2 (CNS2) in the *FoxP3* locus sustaining FOXP3 expression and controlling stable FOXP3 expression inheritance [94]. The requirement for IL-2 in T_reg_ development, homoeostasis and competitive fitness has further been studied in mixed bone marrow chimeric mice co-transplanted with IL-2 signalling-deficient bone marrow and wild-type bone marrow. In these mice, wild-type T_reg_ greatly outnumbered mutant FOXP3^pos^ CD4 T cells in the thymus and in the periphery illustrating the competitive disadvantage conferred by IL-2 signalling deficiency [19, 95]. These experimental designs, however, use bone marrow from IL-2R germline knockout mice and hence, despite analysing thymic as well as peripheral T_reg_, fall short on undoubtedly dissecting the role of IL-2 signalling during development versus its role in homoeostasis of T_reg_. Indeed, the shared signalling pathway with IL-15 and IL-7 and hence their compensatory potential [74] together with data obtained in TCR-transgenic mice [96], indicate that T_reg_ lineage induction can be IL-2 signalling-independent.

### T_reg_-specific rescue of IL-2 signalling

Finally, the intrinsic requirement for IL-2 signalling in T_reg_ maintenance and fitness has been demonstrated in mice with T_reg_ lineage-specific deficiency of CD25 or the IL-2Rβ chain presenting with decreased T_reg_ frequencies and reduced per cell FOXP3 protein levels, and developing fatal autoimmune disease [97]. In line with the aforementioned studies, dysfunctional FOXP3^low^ CD25^neg^ T_reg_ can be found in mice with germline deficiencies of IL-2 and IL-2R [19, 95, 96].

Together, these studies demonstrate the relevance for IL-2 in T_reg_ development, maturation, and survival, and suggest that it serves a direct role in T_reg_ suppressive function.

The role of IL-2 in T_reg_ maturation and function can in part be attributed to a positive feedback loop between FOXP3 and CD25 expression. Upon activation of the IL-2-STAT5 signalling pathway in T_reg_, phosphorylated STAT5 binds the *FoxP3* locus to promote its expression. FOXP3 in turn positively regulates expression of *CD25* [33, 85, 98, 99]. *CD25* expression constitutes part of the T_reg_ transcriptional signature and upon loss of T_reg_ lineage fate, *CD25* gene expression is lost quickly, further illustrating the interdependency of T_reg_ signature genes such as *FoxP3* and *CD25* [100]. High expression of the high-affinity trimeric IL-2R on T_reg_, however, is not only necessary for T_reg_ to scavenge the low levels of IL-2 for their homoeostasis; the ability to preferentially capture IL-2 also presents an immunosuppressive mechanism by starving effector T cells and NK cells from IL-2 and hence limiting their activation and proliferation. The requirement for the IL-2-STAT5-FOXP3 axis in T_reg_ suppressive function was elegantly demonstrated in a study using transgenic mice with T_reg_-specific deficiency of the IL-2R with simultaneous expression of constitutively active STAT5 [97]. Early fatal autoimmune disease otherwise observed in mice with T_reg_-specific IL-2R deficiency can be rescued by constitutive STAT5 signalling; however, the mice still succumbed at a later age from uncontrolled CD8 T cell activation and expansion. This demonstrated that IL-2 consumption via the high-affinity trimeric IL-2R expressed by T_reg_ particularly controls the CD8 T cell population size and activity.

Memory T_reg_ (mT_reg_)—analogous to their non-regulatory counterparts—are long-lived cells which upon secondary exposure however do not respond with proliferation and pro-inflammatory cytokine production but instead possess increased suppressive function. The induction of mT_reg_ would hence be of therapeutic interest. They are thought to mitigate tissue damage during the rapid and heightened response of effector memory T cells upon secondary antigen exposure or to reinforce foetal tolerance during pregnancy. Accordingly, mT_reg_ present as antigen-experienced, CCR7^low^ cells, express high levels of anti-apoptotic BCL-2 and proliferate less (Ki67^low^) compared to activated T_reg_ [101, 102].

While memory T_reg_ may be less dependent on IL-2 for long-term maintenance, they express high levels of CD25 and expand in response to low-dose IL-2 therapy [55, 101, 103–105]. However, long-lived (local) tolerance induced by IL-2-based therapy relies on the pre-existence of antigen-specific mT_reg_ or (local) induction of mT_reg_. Antigen therapy to induce hyposensitivity to allergens might be explained by the induction of mT_reg_. However, e.g. islet-specific antigen therapy alone has been disappointing in trials with type 1 diabetes patients and it has been suggested that antigen therapy must be combined with a T_reg_-inducing agent such as low-dose IL-2. While this presents a promising strategy for type 1 diabetes and other autoimmune diseases, clinical trials are needed to establish antigen dosing and boosting regimens, long-term efficacy and its correlation with mT_reg_ induction and persistence.

The roles of IL-2 in T_reg_ biology and suppressive function make IL-2 a highly attractive immunotherapeutic molecule in the context of autoimmunity and transplantation. However, its activities on different (immune) cell types also demand caution in clinical trial design and close monitoring of adverse effects. In the below chapters, we will discuss recent pre-clinical and clinical efforts to develop IL-2-based immunotherapeutic strategies that target T_reg_ to treat autoimmune conditions characterised by low numbers or reduced suppressive activity of T_reg_ as well as to prevent transplant rejection.

## IL-2-based immunotherapies

IL-2 was the first cytokine therapy approved by the U.S. FDA [58]. The initial indications were in metastatic cancers where IL-2 had to be administered at very high doses (HD IL-2) to achieve clinical benefit. The high doses were necessary because of a very short half-life of IL-2 in vivo and for stimulation the cytotoxic effector T cells and NK cells, presumably, once the high-affinity receptor on T_reg_ has been saturated. The approval was based on the overall objective response rates in up to 20% of patients and durable complete responses for up to 91 months [59]. However, the treatment also induced severe treatment-associated toxicities including vascular leak syndrome and clinical manifestations of a cytokine storm. Efforts to reduce toxicities by lowering the dose led to a considerable loss of therapeutic efficacy due to the expansion of immunosuppressive T_reg_ that contain a high-affinity IL-2 receptor and thereby outcompete other cells for IL-2 [106]. HD IL-2 remains an important treatment in selected patients, either as a first-line option or in combination with new targeted and immunological therapies [107].

The preferential capture of natural IL-2 via the high-affinity IL-2R expressed by T_reg_ is exploited in low-dose (LD) IL-2 therapy. The short half-life of IL-2 (<10 min [108]) requires daily injections of 0.5-3 million international units (MIU) in repetitive treatment courses with effects on T_reg_ lasting days to weeks, but at the same time its quick clearance allows for fast and flexible dose adjustment to ameliorate possible adverse effects. Overall, LD IL-2 treatment is well-tolerated as documented in animal studies and clinical trials (reviewed in [50, 51]). Long-term administration in mice showed no impairment of immune responses or vaccination, nor did it increase cancer occurrence [109]. Similarly, a long-term study in children with early onset type 1 diabetes mellitus (T1D) concluded that the treatment was safe and well-tolerated [110]. However, inherent to the pleiotropic nature of IL-2, dose-dependent mild-to-moderate adverse effects are associated with LD IL-2 treatment. While high-dose IL-2 treatment can induce vascular leak syndrome, LD IL-2 may result in transient influenza-like symptoms, or in eosinophilia driven by ILC2-produced IL-5 [24, 51]. Overall, data obtained in murine disease models and clinical studies are promising with partial or complete response to treatment. Completed and ongoing clinical trials with LD IL-2 in autoimmune and rheumatic diseases are summarised elsewhere [111].

Fuelled by the therapeutic benefit of LD IL-2 and to overcome its limitations, further efforts have focused on the development of second-generation versions of IL-2 with superior pharmacokinetics and T_reg_ selectivity. Aims of these efforts beyond target cell selectivity and reduced off-target effects are to increase the half-life of the novel molecules (at the expense of fast adjustment of dosing in case of adverse effects), less frequent administration, and increased therapeutic dose range. Several groups and pharmaceutical companies have developed PEGylated IL-2 variants [112, 113], IL-2 muteins [114–116], fusion proteins of IL-2 linked to CD25 [117, 118], and IL-2:anti-IL-2 antibody complexes [93, 119–121] that promote T_reg_ cell expansion in vivo.

Here, we will present promising IL-2-based molecules and clinical translation thereof with focus on selected therapeutic IL-2 molecules with post-translational modifications, IL-2 muteins, fusion proteins of IL-2 with other molecules, alternative delivery methods of IL-2, and IL-2:anti-IL-2 antibody complexes (Table 1).

**Table 1:** IL-2-based biologics evaluated for the treatment of autoimmune and inflammatory diseases.

Agent	Mechanism of action	Indication	Treatment (n)	Phase, clinical trial ID, status (completion date)	Biological outcome	Clinical outcome
PEGylated IL-2 variants						
NKTR-358 [126–131]	PEG-IL-2Rα-biased agonist	Healthy volunteers	SAD: eight cohorts (0.3–28.0 μg/kg: *n* = 76; placebo: *n* = 24); subcutaneous; 1st line	Phase 1; NCT04133116, completed (Mar 2020)	Dose-dependent increase in CD4 T_reg_, 12–17-fold increase at the highest dose tested and sustained over 20-30 days; Increased expression of Ki67, HELIOS, CTLA4, ICOS; No significant changes in CD4 and CD8 T_conv_, low level increase in CD56^high^ NK cells.	PK: dose-dependent response and prolonged half-life (mean 7.4–12.9 days); Treatment-related AE primarily limited to mild or moderate injection site reactions; No ADA were detected at any dose or any time point.
		SLE (minimal to moderate)	MAD: four cohorts (3–24.0 μg/kg: *n* = 36; placebo: *n* = 12); biweekly schedule; subcutaneous; 2nd line	Phase 1; NCT03556007, completed (Aug 2019)	Dose-dependent increase in the frequency of CD4 T_reg_, 12–17-fold increase at the highest dose tested and sustained over 20–30 days; Increased expression of Ki67, HELIOS, CTLA4; Hypereosinophilia at the highest doses in some patients, started after the second dose, resolved after treatment discontinuation; No significant changes in CD4 and CD8 T_conv_, low level increase in CD56^high^ NK cells.	AE: mild or moderate (grade 1–2) in severity, primarily injection-site reactions, one patient had flu-like symptoms, 1 patient had moderate hypereosinophilia that resolved after the treatment was discontinued; No ADA were detected at any dose or any time point; No NKTR-358 treatment-related changes in disease activity were apparent as measured by SLEDAI or joint scores.
		Atopic dermatitis	Multiple doses (12 or 24 µg/kg); biweekly dosing; 1st line	Phase 1; NCT04081350, completed (Jan 2023)	Increased total T_reg_ and CD25^bright^ T_reg_ versus placebo during treatment period (12 weeks).	Dose-dependent improvement was observed in disease-relevant scores (EASI, eczema area and severity index) versus placebo up to 36 weeks following end of treatment. No SAEs or severe AEs reported.
		Psoriasis	Multiple doses; 1st line	Phase 1; NCT04119557, completed (Jul 2021)	Increased T_reg_ numbers *versus* placebo during treatment period (12 weeks).	Improved disease score (PASI, psoriasis area and severity index) *versus* placebo, which was maintained up to week 19 post-treatment. Safety profile consistent with previous studies.
		SLE	N/A	Phase 2; NCT04433585, (ISLAND-SLE), completed (Oct 2023)	Dose-dependent proliferation of T_reg_.	The primary endpoint of the study—a four-point reduction in the SLE disease activity index (SLEDAI-2K)—not achieved.
		Ulcerative colitis	Multiple doses; 1st line	Phase 2; NCT04677179, (INSTRUCT-UC), terminated (Aug 2022)	N/A	N/A
THOR-809/ SAR444336 [134]	PEG-IL-2Rα-biased agonist	Healthy volunteers	Single or repeated dose subcutaneous injections; versus placebo	Phase 1; NCT05876767, ongoing (Jul 2023)	N/A	N/A
2021 Zhang et al. [113]	PEG-IL-2Rα-biased agonist	N/A	N/A	Preclinical	N/A	N/A
IL-2 muteins						
Efavaleukin alfa (AMG592) [138–141]	IL-2Rα-biased IL-2m (V91K, C125A) Fc-fusion protein	Healthy volunteers	SAD: (*n* = 6/dose; 8 cohorts) or placebo (*n* = 2/dose) for 28 days; subcutaneous	Phase 1	Dose-dependent T_reg_ expansion; Increased CD25 and FOXP3 in expanded T_reg_; No change in NK cell and minimal increase in T_conv_ numbers; At the highest dose, increase in T_reg_: T_conv_ ratio peaked at day 8 (~4-fold *vs* baseline) and remained elevated up to day 29; No increases in serum proinflammatory cytokines IL-6, TNFα, or IFNγ.	AE: grade 1 painless erythema at/near the injection site which resolved without treatment. PK: dose-related increases in AMG 592 serum exposure.
		SLE	MAD: five dosing cohorts; every 2 weeks; treatment for 12 weeks; subcutaneously; 2nd line	Phase 1; NCT03451422, completed (Oct 2021)	Peak T_reg_ expansion at day 8 post dose sustained for up to 42 days after the last dose; Mean peak increases in T_reg_ 1.1-17.4-fold above the baseline depending on the dose; Increased Helios, PD-1, ICOS, CD39, GITR in expanded T_reg_; No significant changes in CD4 and CD8 T cells nor NK cells; No changes in pro-inflammatory cytokines.	AE: mild-to-moderate injection site reactions, No dose-limiting toxicities; PK: linear and dose-dependent, half-life 18 −30 h.
		RA	MAD: multiple dosing schedules, follow-up for 12 weeks; 2nd line	Phase 1/2; NCT03410056, terminated (May 2020)	N/A	N/A
		GvHD (chronic)	MAD: weekly or every 2 weeks; 2nd line	Phase 1/2; NCT03422627, terminated (Feb 2022)	N/A	N/A
		SLE	Dose-ranging; 2nd line	Phase 2b; NCT04680637, terminated (Jun 2023)	N/A	N/A
		Ulcerative colitis	Dose-finding; 2nd line	Phase 2; NCT04987307, recruiting (Jun 2024)	N/A	N/A
RG7835 (RO7049665) [115]	IL-2Rα-biased IL-2m (T3A, N88D, C125A) IgG1-fusion protein	Healthy volunteers	SAD; subcutaneous	Phase 1; NCT03221179, completed (Jul 2019)	N/A	N/A
		Ulcerative colitis	MAS; subcutaneous; 1st line	Phase 1; NCT03943550, terminated based on the lack of robust clinical improvement in the underlying condition after 8 weeks of treatment (Jul 2021)	N/A	N/A
		Autoimmune hepatitis	Every 2 weeks; subcutaneous administration; 2nd line	Phase 2; NCT04790916, terminated based on a lack of efficacy seen with RO7049665 in a study of ulcerative colitis (Nov 2021)	N/A	N/A
PT101/ MK-6194 [144]	IL-2Rα-biased IL-2m (L118I, N88D, V69A, Q74P, C125S) Fc-fusion protein	Healthy volunteers	SAD: five dose levels from 1 mg to 10 mg, subcutaneous; PT101 (42) or placebo (14)	Phase 1	Dose-related T_reg_ expansion, mean max T_reg_ expansion of 3.6-fold above the baseline on day 8-10 up to day 29; No significant expansion of T_conv_ or NK cells; Transient increases in eosinophil levels in some subjects.	AE grade 1 or 2 and self-limited, most commonly injection-site reactions; No dose-limiting toxicities; PK: peak levels of PT101 11.0–14.6 h after administration, mean half-life 20.4–28.3 h; No ADA detected.
		Ulcerative colitis	MAD, subcutaneous, PT101 or placebo	Phase 1; NCT04924114, recruiting (Feb 2024)	N/A	N/A
DEL106/CC-92252 [146]	IL-2Rα-biased IL-2m (T3A, N88R, C125S) Fc-fusion protein	Healthy volunteers and patients with psoriasis	3-part study: SAD and MAD in healthy volunteers, MAD in psoriasis patients; biweekly dosing for 12 weeks	Phase 1; NCT03971825, terminated due to a lack of progression criteria (Aug 2021)	SAD: Up to 2-fold T_reg_ expansion on day 9-14, max T_reg_/T_conv_ 2-fold above baseline around day 10; No changes in total CD4 and CD8 T_conv_ and NK cells; Increased expression of CD25, FOXP3, CTLA-4, PD-1, ICOS, IL-10 on T_reg_; Increased in vitro suppressive capacitiy of T_reg_ from treated patients MAD: Weekly dosing is not superior to biweekly dosing due to modest T_reg_ increase; MAD in participants with psoriasis: modest T_reg_ expansion in circulation and in skin lesions (up to 2-fold from baseline); No increase in CD4 and CD8 T_conv_ and NK cells; No change in T_H_17 in circulation or lesional IL-17 levels.	MAD in participants with psoriasis: No apparent improvement compared to placebo as measured by target plaque severity score and static physician global assessment AE: mild-to-moderate in intensity, mostly injection-site reactions; Occasional eosinophil and C-reactive protein increases.
CUG252 (ref.^147^, ^148^)	IL-2Rα-biased IL-2m (L19H, C125I, Q126E) Fc-fusion protein	Healthy volunteers, SLE	SAD in healthy volunteers or MAD in SLE patients; 2nd line for SLE patients; subcutaneous	Phase 1; NCT05328557, recruiting (Dec 2023)	N/A	N/A
MDNA209 (ref.^114^, ^149^)	IL-2Rβ-antagonist IL-2m (L18R, Q22E, Q126T, S130R) Fc-fusion protein	N/A	N/A	Preclinical	N/A	N/A
Other IL-2 fusion proteins						
IL-2-CD25 [152, 153]	Human IL-2/CD25 fusion protein	Healthy volunteers, autoimmune diseases	N/A	Phase 1	N/A	N/A
CUE-401 [154, 155]	Human IL-2/TGF-β Fc-fusion protein	N/A	N/A	Preclinical	N/A	N/A
IL2-EHD2-sc-mTNFR2 [157, 158]	IL-2 fused to a TNFR2-selective TNF mutein	N/A	N/A	Preclinical	N/A	N/A
Alternative IL-2 delivery methods						
mRNA-6231 [163]	LNP-mRNA for T_reg_-specific IL-2m/HSA fusion	Healthy volunteers	2-part study: SAD eand MAD; subcutaneous	Phase 1; NCT04916431, completed (Aug 2022)	N/A	N/A
NNC0361-0041 [164]	DNA plasmid encoding PPI, TGFβ, IL-10, IL-2	T1D	MAD in 4 cohorts, early-onset T1D (9) or placebo (3); once weekly for 12w, subcutaneous	Phase 1; NCT04279613, recruiting (May 2024)	N/A	N/A
Viral vector-mediated gene transfer [167]	Viral vector with astrocyte-specific promoter for tissue-specific IL-2 production	N/A	N/A	Preclinical	N/A	N/A
IL-2/mAb complexes						
F5111.2 [120]	Human IL-2/anti-IL-2 mAb	N/A	N/A	Preclinical	N/A	N/A
UFKA-20 [121]	Human IL-2/anti-IL-2 mAb	N/A	N/A	Preclinical	N/A	N/A
Single-chain hIL-2/F5111 mAb-fusion [178]	Single-chain hIL-2/F5111 mAb-fusion protein	N/A	N/A	Preclinical	N/A	N/A

*AE* adverse events, *HSA* human serum albumin, *IL-2c* IL-2/anti-IL-2 antibody complex, *IL-2m* IL-2 mutein, *LNP* lipid nanoparticle, *MAS* multiple ascending dose, *PPI* pre-proinsulin, *SAD* single ascending dose, *RA* rheumatoid arthritis, *SLE* systemic lupus erythmatosus, *T1D* type 1 diabetes, *EASI* eczema area and severity index, *ADA* anti-drug antibodies.

### PEGylated IL-2 variants

PEGylation is a covalent conjugation of proteins to inert polyethylene glycol (PEG) moieties. PEGylation extends the half-life of protein therapeutics by increasing the effective molecular weight of the molecule, while the PEG moieties can also shield the proteins from digestion by proteolytic enzymes via increased steric hindrance. For example, a PEG-modified murine IL-2 increased IL-2 retention in vivo by protection from enzymatic digestion and renal clearance [122]. Although PEG is known as a safe, inert and non-immunogenic synthetic polymer, some FDA-approved drugs are associated with the development of antibodies against PEG moieties that accelerate drug clearance and loss of clinical efficacy [123, 124].

*NKTR-358/LY3471851/rezpegaldesleukin (Nektar/Lilly)* is recombinant human IL-2 (aldesleukin sequence) chemically conjugated with stable PEG moieties, which has an attenuated affinity for IL-2Rβ compared with recombinant human IL-2. NKTR-358 promoted selective T_reg_ activation and increased T_reg_ suppressive function in mice. The durability and specificity of the response was greater following a single subcutaneous administration of NKTR-358 compared to five daily administrations of IL-2, and led to disease suppression in a mouse delayed-type hypersensitivity (DTH) model [125]. Further, biweekly dosing induced preferential and sustained T_reg_ expansion in mice and non-human primates (NHP) resulting in ameliorated disease progression in a mouse model of systemic lupus erythematosus (SLE), and in a non-human primate cutaneous hypersensitivity model [112]. The single ascending dose study in healthy volunteers (NCT04133116) and the multiple ascending dose study with three biweekly subcutaneous doses of rezpegaldesleukin *versus* placebo in patients with SLE (NCT03556007) yielded promising results [126]. Dose-dependent, selective, and sustained increases in percentages and absolute numbers of total CD4^pos^ T_reg_ and CD25^bright^ T_reg_ were observed, with no significant changes in conventional CD4 and CD8 T cells, and low-level increases in NK cells. At the highest dose tested, a 12–17-fold increase in CD25^bright^ T_reg_ over baseline was sustained for 20–30 days. Most adverse events were grade 1–2 injection-site reactions. Immunogenicity was not observed. SLE disease score was not evaluated due to study limitations, however, data for the follow-up phase 1b randomised studies in psoriasis (NCT04119557) and atopic dermatitis (NCT04081350) have been recently presented [127]. Treatment of patients with psoriasis with rezpegaldesleukin resulted in increased T_reg_ numbers, and improved disease score (PASI, psoriasis area and severity index) versus placebo, which was maintained up to week 19 post-treatment [128]. In atopic dermatitis, biweekly subcutaneous injections of rezpegaldesleukin increased total T_reg_ and CD25^bright^ T_reg_ during treatment period (12 weeks), while a dose-dependent improvement was observed in disease-relevant scores (EASI, eczema area and severity index) *versus* placebo up to 36 weeks following end of treatment [129]. Together with a favourable safety profile these data further support clinical development of rezpegaldesleukin in patients with atopic dermatitis [130]. Less encouraging data were reported for phase 2 ISLAND study (NCT04433585) that enroled adults with moderate-to-severe SLE. Although respegaldesleukin led to dose-dependent proliferation of T_reg_, the primary endpoint of the study—a four-point reduction in the SLE disease activity index (SLEDAI-2K)—was not met [131].

*THOR-809/SAR444336 (Synthorx/Sanofi)* is a site-specific PEGylated IL-2 variant with a PEG moiety attached to an unnatural amino acid at the IL-2Rβ interface designed to increase half-life and enhance selectivity for the trimeric IL-2R. The modified IL-2 has a reduced affinity to the IL-2Rβ chain so that the potency of trimeric IL-2R engagement relies on the IL-2Rα chain binding [132]. In mice and NHP, THOR-809 preferentially stimulated proliferation of peripheral T_reg_ relative to effector T cells and NK cells. Expanded T_reg_ had sustained pSTAT5 signalling and upregulated suppression markers FOXP3, CD25, ICOS and HELIOS. Furthermore, THOR-809 administration in mice led to dose-dependent expansion of highly suppressive T_reg_ and control of skin inflammation in the DTH mouse model [133]. A phase 1 trial in healthy subjects (NCT05876767) is currently ongoing [134].

Another promising IL-2 variant with site-specific PEGylation, designated dual 31/51-20 K, similarly displayed substantially increased clearance half-life, preferentially stimulated T_reg_ over effector T cells compared with unmodified IL-2 by selectively reducing the binding affinity for the β subunit of IL-2R, and significantly reduced disease activity and severity in mouse models of xenogeneic graft-versus-host disease (GvHD), SLE and collagen-induced arthritis. Moreover, a single subcutaneous injection of this PEGylated IL-2 did not induce anti-drug antibody formation, nor did it compromise the host defence against viral infection [113].

### IL-2 muteins

The elucidation of the crystal structure of IL-2 bound to its trimeric receptor [11, 135, 136] facilitated the informed introduction of mutations into IL-2 with the aim to increase its affinity or direct its binding. Such targeted mutagenesis allows to uncouple the pleiotropic effects of IL-2 on different immune cells and to target IL-2 activity toward specific cell populations that express either the dimeric IL-2R to boost tumour immunity or the trimeric IL-2R expressed by T_reg_ to increase tolerance in autoimmunity and to transplanted grafts. IL-2 variants with increased binding to CD25 and/or decreased binding to CD122 and/or CD132 preferentially activate and expand T_reg_. These cytokines are further fused to either the fragment crystalisable (Fc) domain of immunoglobulin (IgG) or the full IgG, which results in significantly extended half-life due to increased hydrodynamic radius and hence decreased renal clearance but also due to the recycling of the protein via the neonatal Fc receptor. In the following paragraphs, we discuss a selection of promising IL-2 mutein molecules, each designed for T_reg_ selectivity and application in autoimmune or inflammatory disease, that are currently in clinical development.

*AMG592 or efavaleukin alpha (Amgen)* is an IL-2 mutein with V91K/C125A mutations that confers high CD25-binding affinity, and that is expressed as a fusion to the C-terminus of an Fc homodimer [137]. In a first-in-human study, efavaleukin alpha single subcutaneous administration resulted in dose-dependent T_reg_ expansion, which was highly selective relative to conventional T cells (T_conv_) and NK cells. T_reg_-to-T_conv_ ratio peaked at day 8 (4-fold vs baseline) and remained elevated up to day 29, while no increases in serum proinflammatory cytokines IL-6, TNFα or IFNγ were detected. The expanded T_reg_ displayed increased CD25 and FOXP3 levels and were enriched for CD31^pos^ recent thymic emigrants. Treatment was well tolerated [138] and several early-phase studies were initiated to further evaluate safety and efficacy in subjects with rheumatoid arthritis (RA) (NCT03410056), steroid-refractory chronic GvHD (NCT03422627), and SLE (NCT03451422). Amgen ended the trials in RA due to insufficient therapeutic benefit for the use of efavaleukin alpha plus standard of care therapy in the assessed study population (NCT03410056); and chronic GvHD [139]. Data from a multiple ascending dose phase 1b study in patients with SLE demonstrated that efavaleukin alpha was well tolerated and induced a robust and prolonged dose-dependent T_reg_ expansion, with minimal changes in CD4 and CD8 T_conv_, NK cells or serum levels of pro-inflammatory cytokines [140]. The biweekly administration resulted in a 50-fold increase in CD25^bright^ T_reg_ above baseline, and the T_reg_ numbers remained above baseline for an average of 42 days after the last dose. Despite these promising results, a phase 2b study of efavaleukin alpha in patients with SLE (NCT04680637) has been discontinued as it met pre-defined criteria for futility, i.e., it was unlikely to achieve its objectives [141]. However, a phase 2 study in ulcerative colitis (NCT04987307) is still ongoing.

*RG7835 (Roche)* is a bivalent conjugate of human IL-2 mutein (T3A, N88D, C125A) and a human IgG1 with abolished binding to Fcγ receptors. Due to its reduced affinity to IL-2Rβγ, IgG-(IL-2N88D)_2_ has a 6–9-fold reduced ability to stimulate T_reg_ in human whole blood pSTAT5 activation assays compared to a wild-type IL-2 dimer but had no effect on other cell types except some activity on CD56^bright^ NK cells. Treatment of cynomolgus monkeys and humanised NSG mice (engrafted with human foetal liver CD34^pos^ cells) with a single dose of IgG-(IL-2N88D)_2_ induced sustained 10-14-fold expansion of CD4^pos^ and CD8^pos^ CD25^pos^ FOXP3^pos^ T_reg_ with no effect on other cell types. The in vivo activated and expanded cynomolgus and human T_reg_ had demethylated epigenetic signatures for *FOXP3* and *CTLA4* characteristic of functionally suppressive cells. However, neither mouse disease models nor multiple-dose studies in NHP could be performed due to the immunogenicity of the molecule in both species [115]. Phase 1b study initiated to assess safety, efficacy, pharmacokinetics, and pharmacodynamics of RG7835 in patients with ulcerative colitis (NCT03943550) was terminated after 8 weeks based on the lack of robust clinical improvement in the underlying condition, according to ClinicalTrials.gov. Following the failure in ulcerative colitis, a phase 2 clinical trial designed to evaluate the effect of RG7835 on time to relapse following forced corticosteroid tapering in patients with autoimmune hepatitis (NCT04790916) was also terminated.

Using a structure-guided approach, several mutations in IL-2 were introduced that significantly decreased CD122 binding affinity in addition to other mutations that increased CD25 binding affinity (L118I, N88D, V69A, Q74P, C125S) [142]. The resulting Fc-fusion molecules, *PT101/MK-6194 (Pandion/Merck)*, selectively activated and expanded T_reg_ in preclinical studies in humanised NSG mice and NHP without significant effects on other immune cell types, and without eliciting proinflammatory cytokine production [143]. These expanded T_reg_ had increased expression of FOXP3 and CD25, suggesting enhanced function and stability. In a phase 1a single ascending dose clinical trial in healthy volunteers, PT101 was safe and well-tolerated, and a dose-dependent expansion of CD25^bright^ T_reg_ cells was observed with a mean maximum increase of 72.5-fold for CD25^bright^ T_reg_ by day 8-10 (and an overall 3.6-fold increase in total T_reg_) [144]. T_conv_ and NK cells were not increased while increases in eosinophil counts were transient. A phase 1 clinical trial in ulcerative colitis (NCT04924114) was initiated by Merck & Co. in 2021 to further evaluate PT101/MK-6194.

A similar molecule, an IL-2 mutein (T3A, N88R, C125S) fused to a human IgG Fc domain, *DEL106/CC-92252 (Delinia/Celgene/BMS)* also preferentially binds to IL-2Rα. A single intravenous dose of the compound in cynomolgus monkeys resulted in dose-dependent and selective T_reg_ expansion and activation, which was better compared to IL-2 [145]. An increase in total circulating T_reg_ cells was 15-fold on day 5, while no change in the number of circulating T_conv_ or CD8 cells was detected. The compound also stimulated expression of suppression and proliferation markers CD25, FOXP3 and Ki67 on T_reg_. IL-2 induced selective STAT5 phosphorylation of T_reg_ over a narrow dose range, also activating T_conv_, CD8 T, NK and B cells; in contrast, DEL106 demonstrated over 1000-fold-greater selectivity for T_reg_ over other immune cells. In addition, subcutaneous administration showed that DEL106 exhibited a lower serum clearance and had a longer circulating half-life than IL-2. A phase 1 first-in-human study with this molecule was conducted in three parts: as a single ascending dose or multiple ascending dose study in healthy volunteers and a multiple ascending dose study in psoriasis patients (NCT03971825). CC-92252 was found safe and well-tolerated across studies with adverse effects of mild to moderate intensity. The treatment resulted in a selective but modest (maximum 2-fold) T_reg_ expansion in circulation of healthy participants and in skin lesions of participants with psoriasis. However, as for RG7835 (Roche), no apparent trend of clinical improvement compared to placebo was observed in patients, indicating that the achieved T_reg_ expansion may be insufficient for robust efficacy in active disease. Mechanistic studies revealed that although highly selective, CC-92252 is a weak partial agonist with only a subset of T_reg_ responding to this IL-2 mutein [146]. Given limited evidence for clinical efficacy, the CC-92252 programme has been discontinued, although BMS is pursuing alternative approaches to T_reg_ selectivity with IL-2 constructs (see below, with IL-2/CD25 fusion).

The therapeutic molecule *CUG252 (Cugene/Abbvie)* is an IL-2 mutein (L19H, C125I, Q126E) Fc-fusion protein designed for biased binding activity to IL-2Rα but attenuated binding to the IL-2Rβγ complex [147]. In mice and cynomolgus monkeys, administration of CUG252 resulted in dose-dependent increases in T_reg_ expansion by 10- to 30-fold, with largely abolished activities in effector T cells and NK cells [148]. T_reg_ had enhanced expression of functional and inhibitory markers (CD25, FOXP3, PD-1, CTLA-4, TIM3 and ICOS) and increased suppressive capacity in DTH. In T cell-dependent antibody response models, CUG252 strongly inhibited antigen-driven inflammation, B cell maturation, and antibody production. The molecule is currently in phase 1 study, which aims to evaluate the safety and tolerability of single escalating subcutaneous doses of CUG252 in healthy adult subjects, and multiple escalating subcutaneous doses of CUG252 in patients with mild to moderate SLE (NCT05328557).

*MDNA209 (Medicenna)* is an IL-2 mutein (L18R, Q22E, Q126T, S130R) with increased affinity to the IL-2Rβ and greatly decreased affinity for IL-2Rγ, resulting in attenuated IL-2Rβγ heterodimerization and reduced signalling. The design of MDNA209 is based on the scaffold of IL-2 ‘superkine’ variants that had an increased affinity for the β chain of the IL-2 receptor [114]. Rather than triggering IL-2 signalling, however, MDNA209 acts as an antagonist, blocking the receptor and preventing it from transmitting the signal. When targeted to T cell subsets, this IL-2 variant could be clinically translated in the context of controlling T-cell mediated (auto)immune disorders where it is essential to prevent effector T cell activation and expansion resulting in effector cell-mediated tissue damage, such as during acute GvHD. The mutein and its Fc-fusion version have been characterised ex vivo and in vivo. MDNA209 prevented IL-2- and IL-15-induced signalling *via* STAT5 and blocked proliferation of CD8^pos^ T cells and NK cells, while inhibiting helper T cell type (T_H_) 1, T_H_9 and T_reg_ cells but promoting T_H_17 cell differentiation. Mice treated with an Fc-fusion version of MDNA209 for 10 days showed prolonged survival in a full MHC-mismatched acute GvHD model compared to control IgG [149].

### Other IL-2 fusion proteins

An alternative approach to increase the selectivity of IL-2 for T_reg_ is through fusion with CD25. The *mouse IL-2/CD25 fusion protein* forms a tight inactive dimer that slowly releases the active monomer to stimulate the high-affinity IL-2R [117]. The long-acting biologic expands T_reg_ in vivo more potently than IL-2, but also increases their activation and migration into lymphoid tissues as well as non-lymphoid tissues as shown for the pancreas and its inhibition of anti-insulin autoantibodies. Moreover, the IL-2/CD25 fusion protein was effective in treating diabetes and inhibiting lupus nephritis in mouse models [118, 150]. The human version of the compound is a full agonist, which maintains high selectivity on T_reg_ over other cell types in whole blood pSTAT5 assays [151]. The human IL-2/CD25 had a prolonged half-life and induced a dose-dependent selective increase in T_reg_ in cynomolgus monkeys compared to IL-2 or IL-2 mutein Fc-fusion molecules [146]. The first-in-human study is still ongoing, but preliminary single-dose pharmacodynamics data confirm robust and prolonged T_reg_ induction in humans with no expansion of inflammatory CD8 or T_conv_ cells [152, 153].

*CUE-401 (Cue Biopharma)* is a tolerogenic *IL-2/TGFβ Fc-fusion protein* designed to activate and induce FOXP3 expression in CD4 T cells (iT_reg_). In mouse CD4 T cells, it induces FOXP3 expression in vitro (iT_reg_). Also, in human CD4 T cells from healthy donors, inflammatory bowel disease and RA patients, it results in increased number of FOXP3-expressing cells, however, induction of FOXP3 expression *versus* preferential expansion of containing T_reg_ has not been dissected. The in vitro induced/expanded iT_reg_ suppress polyclonal T cell proliferation and express comparable phenotypic markers as iT_reg_ induced with combination of TGFβ and IL-2 (CD25, CTLA-4, PD-1, GITR, CD38, CD73, GARP). A single dose of CUE-401 administered to TxA23 mice with ongoing autoimmune gastritis increased FOXP3^pos^ CD4 T cells in blood and lymph nodes and inhibited autoreactive T cell proliferation in gastric lymph nodes [154, 155].

TNF signalling *via* TNFR2 enhances expansion, function and stability of T_reg_ [156]. A dual-acting fusion protein, with IL-2 fused to a TNFR2-selective TNF mutein *(IL2-EHD2-sc-mTNFR2)* promoted strong activation and expansion of CD4 and CD8 T_reg_ cells in vitro compared to either IL-2 or TNFR2 stimulation alone, with both components necessary for superior biological activity [157]. The combination of IL-2 and a TNFR2 agonist is therefore a promising approach for selective T_reg_ expansion in vivo [158].

### Alternative IL-2 delivery methods

The therapeutic IL-2 molecules described above are expressed in living cells and are administered as formulations of recombinant protein. Novel technologies are instead based on the in situ expression of encoded proteins and include lipid nanoparticle (LNP)-mediated mRNA delivery, DNA vaccines and gene transfer using viral vectors. Nucleic acid therapeutics are considered safe, well-tolerated and efficacious with major advantages over protein-based therapeutics including simple and cost-effective production processes and opportunities to improve the drug characteristics [159, 160]. However, several challenges remain. The greatest challenge for mRNA nanomedicine is immunogenicity both against the LNP itself as well as against the mRNA-encoded proteins. With gene therapies, which are designed for permanent integration of the viral vector into genome, the uncertainty about delayed adverse events remains the greatest risk factor [160].

*mRNA-6231 (Moderna)* is a lipid nanoparticle (LNP)-encapsulated mRNA encoding a T_reg_ -specific IL-2 mutein fused to human serum albumin (HSA). Two triple-mutant molecules (V69A/Q74P/N88D or V69A/Q74P/V91K) showed the highest difference in pSTAT5 signal between T_reg_ and other cell subsets in human PBMC in vitro and selectively activated and expanded T_reg_ in mice. LNP-formulated mRNA encoding HSA fused to wild-type IL-2 elevated the percentage of T_reg_ in cynomolgus monkeys and was also effective in preclinical models of murine acute GvHD and collagen-induced rat arthritis [161, 162]. The first-in-human trial of mRNA-6231 in healthy adult participants (NCT04916431) was stopped after early clinical data became available [163].

A tolerogenic immunotherapy *NNC0361-0041 (Novo Nordisk)* involves a DNA plasmid which encodes for pre-proinsulin (PPI), TGFβ1, IL-10, and IL-2 [164]. The combination of antigen (PPI) with the three immune response modifiers (TGFβ1, IL-10, and IL-2) is intended to induce antigen-specific T_reg_ accumulating in the pancreas, and to preserve beta cell function in type 1 diabetes (T1D). The safety and efficacy of treatment was demonstrated in NOD mice as assessed by delayed disease progression, necessity of both antigen and IL-2 for increased efficacy and robustness, and tolerability of chronic dosing [165]. However, no pharmacodynamic-related measurements such as T_reg_ activity or cytokine expression were performed. The phase 1 trial in adults with recent-onset T1D is currently recruiting and will evaluate safety, tolerability, and pharmacokinetics of the therapy (NCT04279613).

*Adeno-associated viral (AAV) vector*-mediated gene transfer for systemic and continuous IL-2 production has been investigated using a single administration of an AAV-IL-2 vector in mice. The treatment enabled sustained stimulation and expansion of T_reg_ without inducing effector T cell activation while preventing diabetes in NOD mice [109] or alleviating Alzheimer’s disease in APP/PS1ΔE9 mice with established pathology [166]. Moreover, the long-term IL-2 expression did not impair immune responses to infections, vaccination or cancer [109]. However, this approach does not allow to interrupt or stop the treatment in case of adverse events. A tissue-specific gene-delivery approach of IL-2 for the treatment of neuroinflammatory pathologies has been developed by Yshii et al. T_reg_ constitute a small resident cell population in the brain, where low levels of IL-2 are thought to limit the natural anti-inflammatory processes. Tissue-specific IL-2 expression targeted to astrocytes via an AAV vector induced a local and transient expansion of the T_reg_ cell population in the mouse brain, which led to beneficial effects in mouse models of traumatic brain injury, multiple sclerosis and stroke [167]. Both the tissue-specific IL-2 delivery system as well as the ability to control the encoded protein expression are promising approaches to improve the clinical translation of gene therapy.

### IL-2/anti-IL-2 antibody complexes

Coupling of IL-2 to specific monoclonal antibodies can modify the interaction of IL-2 with its receptor leading to a targeted and longer-lasting in vivo biological activity compared with soluble IL-2 [93, 119]. Depending on the antibody-binding site on IL-2, the IL-2:antibody complex (IL-2c) can preferentially activate either the cells expressing high levels of CD122, such as memory CD8 T cells and NK cells, or CD25-expressing cells such as T_reg_. A prominent and well-studied example is the complex of mouse IL-2 bound to the anti-mouse IL-2 antibody JES6-1. JES6-1 binding sterically obstructs mouse IL-2 interaction with the IL-2Rβγ heterodimer to block the signalling on IL-2Rα^low^ effector cells. Thereupon, IL-2 is preferentially delivered to the trimeric receptor via a unique allosteric exchange mechanism, where the IL-2Rα subunit displaces the JES6-1 antibody allowing IL-2 to initiate signalling via the IL-2βγ subunits. This complex prolonged the in vivo half-life of IL-2 and led to selective expansion of murine T_reg_ in a murine dextran sodium sulphate colitis model [119, 168]. The efficacy of this approach has been further demonstrated in various experimental models of autoimmune diseases or other inflammatory settings as exemplified below:

- enhanced allograft survival in a murine model of islet transplantation and experimental autoimmune encephalomyelitis (EAE) prevention in combination with rapamycin [169],
- markedly attenuated acute GvHD while preserving graft-versus-leukaemia activity after allo-hematopoietic cell transplantation at higher efficacy than tacrolimus treatment [170],
- survival of fully MHC-mismatched skin allograft: IL-2c failed to augment the survival of skin allografts as monotherapy but initial treatment with anti-IL-6 monoclonal antibody followed by supplementation with rapamycin led to graft survival and elevated intra-graft T_reg_ levels [171],
- attenuation of CNS inflammation and neurological deficits in EAE [172],
- suppression of experimental myasthenia gravis [173],
- inhibition of collagen-induced arthritis [174],
- attenuation of atherosclerosis in apolipoprotein E-deficient mice [175],
- decreased myofiber injury in murine muscular dystrophy model [176].

These results motivate the investigation of IL-2-based therapies in inflammatory diseases or conditions that are not caused by autoimmune or alloimmune reactions.

A fully human anti-IL-2 antibody *F5111.2* that resembles the exchange mechanism observed for the anti-mouse IL-2 antibody JES6-1, was developed by Trotta et al. [120]. Comparison of the crystal structure of IL-2c with the IL-2/IL-2R quaternary structure revealed that F5111.2 sterically obstructs the binding of human IL-2 to IL-2Rβ and allosterically reduces the affinity of the cytokine to IL-2Rα. Administration of F5111.2-hIL-2 complex results in the preferential STAT5 phosphorylation of T_reg_ in vitro and selective expansion of T_reg_ in vivo. When complexed with human IL-2, F5111.2 induced remission of T1D in the NOD mouse model, reduced disease severity in a model of EAE and protected mice against xenogeneic GvHD [120].

Another anti-human IL-2Rα-biased IL-2 antibody, *UFKA-20*, uses a similar mechanism to selectively target T_reg_ [121]. The IL-2 bound to UFKA-20 fails to induce cell activation via the dimeric IL-2R unless the cells also express CD25. Once the IL-2/UFKA-20 complex is bound to CD25, the antibody dissociates from IL-2 and allows the formation of high affinity quaternary IL-2/IL-2R structure that leads to intracellular signalling. Consequently, the IL-2/UFKA-20 complexes efficiently and preferentially stimulated CD4^pos^ T_reg_ in freshly isolated human T cells ex vivo and in mice and rhesus macaques in vivo [121].

The clinical translation of the IL-2/antibody complex approach is complicated by the instability of the cytokine/antibody complex and the need to optimise dosing ratios, as dissociation would lead to off-target effects and rapid clearance. Genetically fusing IL-2 and the antibody should circumvent these drawbacks [168, 177]. A *single-chain hIL-2/F5111 antibody-fusion* protein has been engineered that demonstrated selective T_reg_ bias and showed efficacy in mouse models of colitis and checkpoint inhibitor-induced diabetes mellitus [178].

## Conclusions

IL-2 is central in the biology of T_reg_ during development, functional maturation, lineage stability, peripheral homoeostasis, and function. The consequence of the dependency of T_reg_ on IL-2 is the development of autoimmunity in the absence of IL-2 signalling. T_reg_ compensate for the dependency with an exceptional IL-2 capture sensitivity that outcompetes that of other cell types. The necessity for IL-2 signalling and the high expression of the high-affinity trimeric IL-2R make the IL-2 signalling pathway a prime-candidate for T_reg_-targeting therapeutic approaches in autoimmune and inflammatory diseases as well as in the prevention of transplant rejection.

Despite its high efficacy, given the limitations of low-dose IL-2, numerous approaches have been developed to increase the targeting specificity of IL-2 and hence to avoid binding of the new IL-2-based biologicals to non-T_reg_ cells. Informed by structural and empirical studies, modified IL-2-based molecules are being tested in pre-clinical studies as well as in clinical trials. Yet, informed design may not entirely predict therapeutic success as illustrated by insufficient efficacy and incomplete translation of pre-clinical data in clinical trials for some candidates. However, despite the requirement for thorough clinical assessment of therapeutic benefit in each disease, recent successes in clinical trials for several modified IL-2-based molecules in various autoimmune contexts are representative of the promising therapeutic perspective of IL-2-based immunotherapeutics.

The further possibility to target (modified) IL-2 to T_reg_ subsets of particular prevalence in a disease context by the use of additional moieties may expand the drug development toolbox in the future. Similarly, combinatorial therapy, such as with rapamycin, may prove beneficial but will require assessment in clinical trials. Finally, and undoubtedly, an increasing understanding of structural modifications and their functional consequences will further the design of IL-2-based molecules to increase targeting efficiency as well as to minimise risk for off-target activity and hence maximise safety and efficacy.
